# A Systematic Review with Meta-Analysis on the Effects of Plyometric-Jump Training on the Physical Fitness of Combat Sport Athletes

**DOI:** 10.3390/sports11020033

**Published:** 2023-01-30

**Authors:** Alex Ojeda-Aravena, Tomás Herrera-Valenzuela, Pablo Valdés-Badilla, Eduardo Báez-San Martín, Rohit K. Thapa, Rodrigo Ramirez-Campillo

**Affiliations:** 1 IRyS Group, Physical Education School, Pontificia Universidad Católica de Valparaíso, Valparaíso 2581967, Chile; 2 Department of Physical Activity, Sports and Health Sciences, Faculty of Medical Sciences, Universidad de Santiago de Chile (USACH), Santiago 9170022, Chile; 3 Department of Physical Activity Sciences, Faculty of Education Sciences, Universidad Católica del Maule, Talca 3530000, Chile; 4 Sports Coach Career, School of Education, Universidad Viña del Mar, Viña del Mar 2520000, Chile; 5 Department of Sport Sciences, Faculty of Physical Activity and Sport Sciences, Universidad de Playa Ancha, Valparaíso 2340000, Chile; 6 School of Physical Education and Sports, Rashtriya Raksha University, Gandhinagar 382305, India; 7 Exercise and Rehabilitation Sciences Institute, School of Physical Therapy, Faculty of Rehabilitation Sciences, Universidad Andres Bello, Santiago 7591538, Chile

**Keywords:** plyometric exercise, human physical conditioning, resistance training, muscle strength, musculoskeletal and neural physiological phenomena, musculoskeletal physiological phenomena, exercise, sports science, sports medicine, athletic performance

## Abstract

We aimed to assess the athletic performance changes in combat sport athletes (CoSAs) after plyometric-jump training (PJT), compared to control conditions, through a systematic review with meta-analysis. Following PRISMA guidelines, three electronic databases were searched for includable articles, according to a PICOS approach. Using a random-effects model, Hedges’ g effects sizes (ES) were calculated. Heterogeneity was assessed using the I^2^ statistic, with values of <25%, 25–75%, and >75% representing low, moderate, and high levels of heterogeneity, respectively. Statistical significance was set at *p* ≤ 0.05. The certainty of evidence was assessed using the GRADE approach. Twelve eligible articles were identified for systematic review, seven of high quality and five of moderate quality, according to the PEDro scale. The studies recruited taekwondo, silat, wrestling, judo, fencing, and karate athletes (292 total participants), including specific–active and active controls. Most participants had a mean age of <18 years and were males (*n* = 225). Compared to the control, PJT programmes, involving 4–12 weeks and 2–3 sessions per week, induced small to moderate improvements (ES = 0.47 to 1.04) in athletes’ maximal strength (e.g., 1RM squat), vertical jump height, change-of-direction speed, and specific performance (e.g., fencing movement velocity), although without meaningful effects on body mass, fat mass, and muscle mass (ES = 0.02 to −0.06). Most (7 of 8) outcomes attained low heterogeneity. The outcome-level GRADE analysis indicated a certainty of evidence from low to moderate. In conclusion, PJT, when compared to control conditions, may improve CoSA athletic performance.

## 1. Introduction

Combat (contact) sports typically entail one-on-one combat between competitors under a specific ruleset [1], involving disciplines that are highly popular worldwide, which is consistent with the increase in the number of published papers on the subject in recent years [2,3]. Combat sports can, typically, be classified into percussive sports (i.e., karate, taekwondo, boxing, fencing) or dominance sports (i.e., wrestling, judo) [4]. Among these combat sports, many are part of the Olympic games [5,6], such as boxing, wrestling, fencing, judo, taekwondo, and karate. Other combat sports, such as Brazilian jiu-jitsu and mixed martial arts, are popular and expanding [7].

Of note, combat sports are characterized by posing high demands on the athletes’ physical fitness [8,9,10]. Indeed, the competitive level of combat sport athletes (CoSAs) might be differentiated according to their athletic performance. For example, standing long jump performance (*p* = 0.03) and 10 × 5 shuttle run performance (*p* < 0.001) were higher in elite vs. sub-elite athletes, and 10 × 5 shuttle run performance was related to competitive success (R^2^ = 0.221; *p* = 0.006) in female karate athletes [11]. Further, one-repetition maximum (1RM) and muscle power in upper and lower extremities predicted 89.1% achievement of elite level, and Wingate test (crank–arm) peak power predicted male wrestling success (odds ratio = 0.987; *p* = 0.001) [12]. In addition, body composition can be related to jiu jitsu athletes’ performance levels [13,14,15].

Therefore, it is important that CoSAs implement optimal training activities to develop athletic performance components associated with success (e.g., muscle strength and power, agility, and body composition) [16,17,18,19,20,21,22]. Several supplemental training methods are routinely used by CoSAs to optimize their athletic performance [16,17,18,19,20,21,22,23,24,25,26]. Among these, plyometric-jump training (PJT) can induce significant benefits [27,28,29,30,31,32,33,34] in muscle strength [35], power [36], and body composition [28,30]. PJT exercises involve the use of rapid eccentric and concentric muscle–tendon actions (i.e., stretch–shortening cycle), with jump exercises involving shorter (e.g., <250 ms) or longer (e.g., ≥250 ms) ground contact times and maximal jump height/distance (i.e., reactive strength index) as distinctive markers of performance during training sessions [37]. These PJT exercises promote a series of physiological and biomechanical responses (e.g., high rate of force development) that can lead to improved athletic performance [27,37,38,39,40]. Indeed, PJT induces neuro-mechanical adaptations [27], with high transference to specific CoSA performance [41,42,43]. For example, experienced male fencers (aged ~25 y) improved their fencing movement times after 12 weeks of PJT combined with resistance training [41]. Similarly, highly trained karate athletes (aged ~22 y) applying PJT for 6 weeks, having 2 sessions per week, experienced noted improvements in physical fitness, and in markers of injury risk [42]. Moreover, the maximal strength of young (aged ~17 y) male silat athletes improved after 6 weeks of PJT [43]. Further, after 6 weeks of PJT intervention among young (age, 17.8 y) male fencers, PJT induced similar improvements in physical fitness (in 13 of 19 measures) when compared to accentuated eccentric training [44]. However, the numbers of CoSAs participating in PJT studies are usually ten or less per group [37,45,46], precluding robust conclusions [47]. Moreover, contrasting findings have been reported regarding the athletic performances of CoSAs after PJT [10,48].

To address the aforementioned limitations, a systematic review with a meta-analysis approach can offer relevant advancement in the field [49]. Such an approach also allows the detection of gaps and limitations in the literature, thus providing future research avenues to researchers. Thus, our main aim was to assess the athletic performance changes in CoSAs after PJT, compared to control conditions, through a systematic review (with meta-analysis). We hypothesized that PJT would improve the physical fitness and specific sports abilities of CoSAs compared to controls.

## 2. Materials and Methods

### 2.1. Literature Search, Administration, Update, and Inclusion and Exclusion Criteria

A systematic review was conducted following international standards (i.e., PRISMA guidelines) [50,51], including specific recommendations in the field of PJT [37,46]. Briefly, a systematic scoping review started on April 2017, with updates until November 2022. The search strategy for the databases PubMed, Web of Science, and SCOPUS, and the background of the search history, are described in Table 1.

One researcher (RRC) oversaw identification and screening processes. At the eligibility stage the PICOS approach [50] was considered (Table 2). Additional exclusion criteria have been previously detailed [37,46]. Briefly, we excluded documents classified as books or book chapters, congress abstracts, cross-sectional studies, reviews, and training-related studies without a focus on PJT exercises (e.g., upper body plyometrics). The researchers RRC and PVB individually read and confirmed the eligibility inclusion of full-text studies, with a third author (THV) providing arbitrage, if necessary. Other potentially relevant studies were searched in the lists of references in the included studies.

### 2.2. Data Extraction

Relevant athletic performance attributes for CoSAs [52,53,54,55,56,57,58,59,60,61,62] were considered for data extraction, including 1RM in squat (maximal dynamic strength), vertical squat and countermovement jump height, change-of-direction speed (CODS), body mass, fat and muscle mass, and CoSA specific performance (e.g., fencing movement velocity). These outcomes were considered reliable [63,64,65,66], a key element for meta-analysis [50]. If needed, a valid software [67] was used to extract data from studies that presented results only in figure format.

### 2.3. Studies Methodological Quality

A valid and reliable tool (i.e., PEDro scale) [68,69,70] assessed the studies in eleven dimensions, with ten of these receiving a punctuation for quality assessment, as in previous PJT studies [37,71,72]. Some PEDro scale items (e.g., blinding of participants) [73] are difficult to accomplish in PJT interventions. Therefore, following previous recommendations [33,71,74] the studies were assessed as with “poor”, “moderate”, or “high” quality if theses achieved ≤3 points, 4–5 points, or 6–10 points. Two authors (AOA and EB) independently assessed/confirmed the quality of the studies, with a third author (PVB) providing arbitrage, if necessary.

### 2.4. Meta-Analyses

Although meta-analyses can be performed with 2 studies [75], we considered it more appropriate to perform meta-analyses when at least 3 studies were available for a given outcome [60,76], which was a particularly relevant consideration when taking into account the low number of participants usually involved in PJT studies [37,46,47,77,78]. Using a random-effects model [79,80], the Hedges’ g effect sizes (ES) were calculated (Comprehensive Meta-Analysis software; version 3, Biostat, Englewood, NJ, USA) for the included dependent variables, reported with their confidence intervals (95% CIs), and assessed as trivial, small, moderate, large, very large, and extremely large, for values <0.2, 0.2–0.6, >0.6–1.2, >1.2–2.0, >2.0–4.0, >4.0, respectively [81]. If a given study included one control group and two or more experimental groups in the meta-analysis, the control group sample size was proportionally divided as per the number of experimental groups [82]. The I^2^ statistic was used to assess heterogeneity, with values of <25%, 25–75%, and >75% representing low, moderate, and high heterogeneity, respectively [83]. The extended Egger’s test assessed risk of publication bias [84,85,86] for outcomes with 10 or more studies, and, thereafter, the trim and fill method was used [87], considering a default estimator (L0) for the number of missing studies [88]. When *p* ≤ 0.05, the significance was considered for statistical analyses.

### 2.5. Moderator Analyses

The moderator analyses were planned for outcomes with six or more sub-groups to compare (e.g., female vs. male; taekwondo vs. karate; less than eight weeks of PJT compared to more than eight weeks of PJT). When appropriate, the median split technique [89,90,91] was used for sub-group allocation.

### 2.6. Certainty of Evidence

Two authors (RRC and PVB) assessed/confirmed outcome-level certainty of evidence according to the GRADE recommendations [92,93,94,95].

## 3. Results

### 3.1. Studies Selection, Inclusion, and Quality Assessment

**Figure 1:**
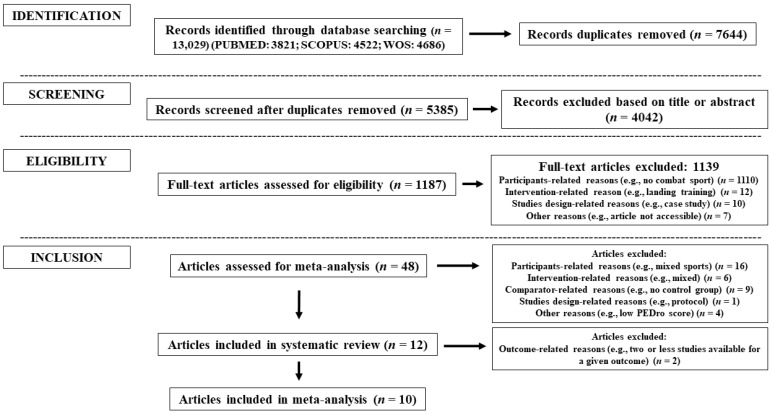
Search process flow diagram.

Figure 1 provides a flow chart illustrating the study selection process. Twelve studies were considered eligible for systematic review [10,41,43,44,45,48,96,97,98,99,100], although two were not included in meta-analyses [98,99]. Most studies (*n* = 7) attained a high PEDro score (≥6 points), although no study scored >7 points (Table 3).

### 3.2. Study Characteristics

The 12 studies included in the systematic review recruited taekwondo, silat, wrestling, judo, fencing, and karate athletes (Table 4). Most participants (*n* = 225, [76.8% of total participants]) were males, and 10 studies recruited youth participants (aged <18 years). Among control groups, two were specific–active controls [44,98] participating in either accentuated eccentric or balance training, while the remaining controls participated in their standard CoSA routines. The PJT interventions lasted 4–12 weeks, with 2 or 3 weekly sessions (Table 4).

### 3.3. Meta-Analyses

The findings derived from the meta-analyses are presented in Table 5. Briefly, the PJT interventions induced significant improvements (all *p* < 0.05) in CoSA physical fitness, when compared to control conditions, including maximal dynamic strength, jumping performance (CMJ), CODS, and CoSA specific performance. When compared to control conditions, no significant effect (*p* > 0.05) of PJT was noted on CoSA body composition (muscle mass, fat mass) or body mass.

In Table 6 it can be seen that the GRADE analysis indicated that CMJ results attained a low certainty of evidence. However, the rest of the results achieved a moderate grading.

## 4. Discussion

### 4.1. Main Findings

The main aim in this systematic review with meta-analysis was to assess the athletic performance changes in CoSAs after PJT, compared to control conditions. A total of 12 studies were included in this systematic review, and 10 in the meta-analyses, involving 293 taekwondo, silat, wrestling, judo, fencing, and karate CoSAs (mostly males) with a mean age between 11 and 25 years. From small (ES = 0.47) up to moderate (ES = 1.04) improvements were noted in the physical fitness of CoSAs after PJT, when compared to control conditions, including 1RM in squat (dynamic maximal strength), lower body power (vertical jump performance), CODS, and CoSA specific performance (e.g., fencing movement velocity), usually after 4 to 12 weeks of intervention, and with two up to three weekly training sessions. However, body mass, fat and muscle mass were not affected by PJT, when compared to control conditions (ES = 0.02 to −0.06).

### 4.2. Athletic Performance

Interventions involving PJT exercises demonstrated significant transference effect of physiological–biomechanical adaptations to sport-specific performance in soccer (e.g., kicking velocity) [101,102,103], water-sport athletes [104], and endurance runners [31]. The results derived from this meta-analysis supported previous findings, adding a novel contribution regarding the transfer of PJT-induced adaptations toward CoSA specific athletic performance, providing high-level evidence-based information that may support practitioners’ decisions when designing training schedules [49]. The improvements following PJT may be attributed to different neuromuscular adaptations that result in an increase in the rate of force development, in line with the maximal level of force that an athlete can generate. These improvements may be plausible due to the physiological adaptations through PJT, that includes (but is not limited to) increased muscle fibre force, velocity and power capability, and electromyography activity (e.g., number of motor units recruited and recruitment rates of motor units) [27,35,105,106,107,108]. Indeed, maximal-intensity short-duration PJT efforts may mimic the physiological–biomechanical demands (e.g., fast-force production capabilities) of the efforts in CoSA competitions (e.g., punch and kick) that may be key elements of success [16,17,18,19].

Furthermore, these same neuromuscular adaptations may be related to improved maximal strength (e.g., 1RM back squat), vertical jumping, and CODS performance noted after PJT [27,36,72]. However, the improvements may also be related to the skeletal muscle hypertrophy attained through PJT [30,109]. Indeed, PJT is reported to induce similar hypertrophy in lower limb muscles as that of resistance training [30], thus implicating the importance of PJT among CoSAs. In addition, another adaptation responsible for the improvements in physical fitness abilities is increased muscle–tendon stiffness (e.g., Achilles tendon stiffness) through PJT [110]. The increase in Achilles tendon stiffness may be attributed to the eccentric loading of the musculotendinous unit during various PJT exercises, and may lead to improved transfer of eccentric and concentric force of the muscles (e.g., knee extensors), and, thus, improving the performance of physical fitness abilities. The muscle–tendon adaptations also improve the reactive strength of the lower limbs which is associated with various physical and sports-specific performances [111]. Relatedly, improved dynamic maximal strength, jumping and CODS performance may also play a relevant role in sport-specific performances in CoSAs [11,12].

### 4.3. Anthropometric Adaptations

In addition to improved physical fitness abilities, CoSAs also need optimal body composition to maximize performance [16,17,18,19]. Interventions involving PJT exercises are capable of inducing increases in muscle mass and reductions in body fat [28,30]. However, when control conditions were compared to PJT in the current meta-analyses, no changes were noted for total body mass, fat mass, and muscle mass. However, a lack of body mass changes may be considered a positive result for CoSAs. Indeed, an increased muscle power (e.g., greater jumping ability), in line with unaltered body mass, would increase the power relative to the athlete’s total body mass (i.e., W.kg^−1^), a key determinant for CoSA success [16,17,18,19]. Regarding muscle mass, PJT may induce muscle hypertrophy effects [28,30,109]. Nonetheless, there was probably an insufficient time period (i.e., six weeks) to induce detectable hypertrophy in the studies providing data for muscle mass after PJT that were included in our meta-analysis [112]. For adipose markers, a relatively high jumping rate (e.g., 100 per minute) with reduced, or no, inter-repetition rest, can increase cardiorespiratory responses [113,114], and, thus energy expenditure and so, potentially, contributes to reduced fat mass [115]. However, in the studies analyzed in the current meta-analysis, traditional PJT interventions were incorporated, usually involving a lower amount of total energy expenditure [28,37,46]. This may partially explain the lack of changes in body fat. Nevertheless, the diet was not controlled in studies. Future studies may seek to address this relevant shortcoming in the literature.

### 4.4. Limitations

Firstly, the moderator analyses, and the risk of publication bias analyses were precluded, due to the reduced number (i.e., less than six) articles available for each outcome. Similarly, a mean of only 11 participants were included per study group. Secondly, the analyzed studies failed to report relevant methodological information, such as the intensity used during PJT exercises. Finally, more robust evidence is needed before definitive conclusions regarding the optimal dose of PJT for CoSAs. Future researchers should include larger numbers of participants for both control and experimental groups, involving appropriate randomization procedures, and adequate reporting of meaningful physical fitness variables that can be potentially improved with PJT (e.g., endurance).

### 4.5. Practical Applications and Future Lines for Research

The available evidence suggests that the incorporation of PJT may be effective in both male and female, and in youth and adult, CoSAs, independent of their previous experience with PJT, specific combat sport practiced, competitive level, or period of the season (e.g., in-season vs pre-season). A minimal effective dose of PJT may involve 2 weekly sessions, for 4 weeks. Although the intensity is difficult to prescribe, high-maximal exercise seems safe if adequate technique and progression are considered by the professionals in charge of the training sessions. Different types of jumps can be incorporated, and be regarded as effective, such as CMJ, DJ, horizontal jumps, repeated jumps, rope jumps, loaded jumps, single-leg jumps, and lateral–diagonal jumps. When DJ is used, a minimal effective box-drop height of 15 cm may be considered, and up to 50 cm. The number of jumps per session may vary greatly depending on factors such as the type of exercise and its intensity. For example, for jumps such as rope jumps (like repeated CMJ) 3 min per session may be regarded as an effective initial dose. Assuming a jump rate of 100 jumps per minute, 3 min of jump rope would involve 300 jumps per session. For loaded jumps, such as jump squats, as low as 9 repetitions per session may represent a minimal effective initial dose. The recovery time may also depend on factors such as the type of PJT exercise, intensity, and CoSA characteristics (e.g., youth athletes may recover faster than adults) [116]. Between repetitions the recovery time may be minimal, and, if applied, this probably should be no more than 15 s [117]. Between-sets recovery of ≥30 s should probably be considered, and between-sessions recovery periods of ≥48 h may be effective. Progressive overload is a basic training principle and probably should be considered by CoSAs for long-term PJT programming optimization [118,119,120,121,122], either as volume-based, technique-based, or type of exercise-based, or as a mixture of these overload-based techniques.

Of note, although PJT may be effective when applied in isolation, from a practical standpoint, potentially greater effects can be expected when PJT is applied in a multi-component training program [23,123,124], in line with the objectives of long-term physical development strategies [118,119,120,121,122]. Of note, four studies in our meta-analysis [41,48,99,100] involved a combination of PJT with another type of exercises, such as traditional (high-load slow-velocity) resistance training. Although a moderator analysis was not considered, due to the reduced number of available studies for each moderator category (i.e., combined vs non-combined), the studies that applied a combined training approach (Table 4) attained a meaningfully greater CMJ improvement (ES = 1.3), compared to those that only focused on PJT drills (ES = 0.2). In fact, in *real-world* settings, when PJT is introduced to CoSAs, this should be combined with the training methods that athletes regularly use. In this context, one study [125] indicated that it may be advantageous for PJT to be incorporated at the beginning of regular training sessions rather than at the end. Indeed, movements involving fast SSC tendon–muscle action may be hindered after a regular training session [126], reinforcing the idea of embedding PJT at an early stage of CoSA training sessions (e.g., after warm-up). Therefore, potentially greater effects in physical fitness may be expected if PJT is applied without fatigue [127]. Avoidance of fatigue before PJT exercises may be relevant, especially if PJT exercises involve high-intensity movements (e.g., high eccentric force and short ground contact times) [128]. Further, to avoid excessive fatigue, if PJT is incorporated into a CoSA’s regular training schedule, it could be effectively included even if a portion of the athlete’s standard routine is replaced, which may help to reduce the risk of overtraining–overreaching. Additionally, before important competitions, a tapering approach (usually as reduced number of jump repetitions) may be useful to reduce potential fatigue before competitions.

Regarding participants’ sex, only three studies involved females (Table 4) (~23% of total participants). The lower number of females compared to males is, unfortunately, relatively common in the PJT literature [37]. The reason why females are less involved in PJT research is probably multifactorial and not only related to PJT but, overall, to strength and conditioning research [129,130,131,132]. Likely reasons could be that, for many years, fewer females practiced professional sports (e.g., soccer, track and field) that benefited from PJT, compared to males. From a global perspective, cultural and/or religious reasons may even reinforce this phenomenon, particularly for CoSAs. In addition, PJT, and power exercises in general, may not have been in the scope of the approaches considered by coaches in the context of exercising either females or males. The positive effects of PJT exercises for females could be less well known among coaches, and researchers have neglected this topic for many years, only extending their research efforts to include females just recently. There is evidence [133] that it takes up to 17 years until research findings are translated into (clinical) practice. Such a limitation is applicable to studies regarding athletes as well, such as regards female CoSAs. Indeed, in the current systematic review, most (10 of 12) of the studies that recruited athletes included males. With the increased participation of females in sports, research is required to enhance knowledge with regards to PJT programming for female athletes. The number of female athletes involved in sports, as well as the number of studies conducted within the female population and among female athletes, is steadily increasing [129].

Future studies may assess the potential effect of PJT moderators, such as the type of surface, tapering, and exercise intensity, particularly using interventions with a relatively longer duration [27,40,134]. Moreover, PJT tapering may be a relevant strategy to consider in future studies [135,136,137,138] in CoSAs, particularly regarding weight reduction before competition. Further, 9 of the 12 studies analyzed involved fencing (*n* = 5) and taekwondo (*n* = 4) athletes. Therefore, more research is needed regarding some specific CoSAs, as it is difficult to simply translate the results from one specific combat sport to another, due to their different physiological–biomechanical demands [16,17,18,19]. Indeed, the magnitude of the PJT effect may be different according to the participants’ sports background [139,140]. In fact, one of the included studies in our meta-analysis noted that outcomes, such as contact time, relative leg stiffness, and reactive strength index, may respond differently to PJT, depending on the athletes’ sports backgrounds (i.e., taekwondo vs. rhythmic gymnastics) [10]. However, such phenomena may depend on the outcome being analyzed. For example, the magnitude of the PJT effect may be similar for maximal strength and jump performance, independent from sports background [35,141]. This was corroborated in a meta-analysis where similar improvements in jump performance were noted for gymnasts (ES = 0.51), runners (ES = 0.40), and athletes from mixed sports, including CoSAs (ES = 0.57) [142]. Further, similar improvements in maximal strength performance were noted for runners (ES = 0.51) and mixed sports that included CoSAs (ES = 0.46) [142]. Future studies should determine the role of sports background on the adaptation of CoSAs to PJT.

Of note, most of the included studies in our systematic review involved moderately trained CoSAs. Indeed, in our systematic review we considered previous recommendations [37] to categorize CoSA fitness level. The category “high” was considered for professional/elite athletes with regular enrolment in national and/or international competitions, highly trained participants with ≥10 training hours per week or ≥6 training sessions per week and regularly scheduled official-friendly competitions. The category “moderate” was considered for non-elite/professional athletes, with a regular attendance in regional and/or national competitions, between 5–9.9 training hours per week or 3–5 training sessions per week and regularly scheduled official and friendly competitions. Accordingly, future studies are needed in highly trained and professional CoSAs.

## 5. Conclusions

The current systematic review, that included 12 controlled articles, and the meta-analyses, that included 10 articles, indicated that CoSAs may achieve athletic performance improvement after PJT, including improvements in maximal strength, jumping, CODS, and sport-specific performance, without alterations in their body compositions.

## 6. Registration

Open Science platform (OSF), under the registration doi 10.17605/OSF.IO/NWHS3. Some adaptations were incorporated posteriori to adapt the protocol for the specific characteristics of combat sport athletes.

## Figures and Tables

**Table 1:** Search strategy (code line) for each database and background of search history.

**Date of the search**	April, 2017	May, 2019	August, 2021
**Databases**	PubMed	PubMed, WOS (Core Collection), Scopus	PubMed, WOS (Core Collection) ^a^, Scopus
**Keywords**	“plyometric”, “training”	“ballistic”, “complex”, “cycle”, “explosive”, “force”, “plyometric”, “shortening”, “stretch”, “training”, “velocity”	“ballistic”, “complex”, “cycle”, “explosive”, “force”, “jump”, “plyometric”, “power”, “shortening”, “stretch”, “training”, “velocity”
**Database fields for the search**	All	PubMed: all WOS: all Scopus: title, abstract, keywords	PubMed: all ^b^ WOS: all ^b^ Scopus: title, abstract, keywords ^b^
**Restrictions for the search**	None	None	None
**Examples of search strategy code line**	-PubMed: “plyometric exercise”[MeSH Terms] OR (“plyometric”[All Fields] AND “exercise”[All Fields]) OR “plyometric exercise”[All Fields] OR (“plyometric”[All Fields] AND “training”[All Fields]) OR “plyometric training”[All Fields] -WOS: (ALL = (plyometric)) AND ALL = (training) -SCOPUS: TITLE-ABS-KEY (plyometric AND training)		

^a^: except for the keywords “jump” and “power” searched in all WOS databases; ^b^: except for the keywords “jump” and “power” searched in the database field TITLE (a very poor efficiency was obtained in the search for results with the incorporation of other database fields); Note: after the formal database search, the list of included articles and the inclusion criteria (see Table 2) were sent to three independent world experts in the field of physical fitness and sport-specific performance, plyometric jump training, and combat sport athlete (https://www.expertscape.com/ex/physical+fitness (accessed on 8 November 2022); https://www.expertscape.com/ex/plyometric+exercise (accessed on 8 November 2022); https://expertscape.com/go/martial%20arts (accessed on 8 November 2022)) to help identify additional relevant articles. Additionally, the experts had peer-reviewed publications in the fields of physical fitness and sport-specific performance, plyometric jump training and/or combat sport athlete. The experts were not provided with our search strategy, to avoid biasing their own searches. Upon completion of all these steps, the databases were again consulted in a search for any errata or retractions in any of the included studies.

**Table 2:** Selection criteria used in the systematic review.

Category	Inclusion Criteria	Exclusion Criteria
Population	Healthy combat sport athletes, with no restrictions on their fitness or competitive level, sex, or age.	Participants with health problems (e.g., injuries, recent surgery), precluding participation in a plyometric jump training program.
Intervention	A plyometric jump training program, with a minimal duration of ≥3 weeks, which included unilateral and/or bilateral jumps, which commonly utilize a pre-stretch or countermovement stressing the stretch–shortening cycle.	Exercise interventions not involving plyometric jump training (e.g., upper body plyometrics only training interventions) or exercise interventions involving plyometric jump training programs representing less than 50% of the total training load (i.e., volume, e.g., number of exercises) when delivered in conjunction with other training interventions (e.g., high-load resistance training).
Comparator	Control group (i.e., standard sport training; alternative training intervention; physically active; non-active).	Absence of control group.
Outcome	At least one measure related to physical fitness (e.g., countermovement jump height; body fat) and/or sport-specific performance (e.g., kicking speed) before and after the training intervention.	Lack of baseline and/or follow-up data.
Study design	Multi-arm trials.	Single-arm trials/observational studies.

**Table 3:** Scores derived from the PEDro rating scale.

	1	2	3	4	5	6	7	8	9	10	11	Score ^a^	Study Quality
Akın & Kesilmiş, 2020 [45]	1	0	0	0	0	0	0	1	1	1	1	4	Moderate
al Syurgawi & Mohamed Shapie, 2019 [43]	1	1	0	1	0	0	0	1	1	0	1	5	Moderate
Chaouachi et al., 2014 [96]	1	1	0	1	0	0	0	1	1	1	1	6	High
Dallas et al., 2020 [10]	1	1	0	0	0	0	0	1	1	1	1	5	Moderate
di Cagno et al., 2020 [44]	1	1	0	1	0	0	0	1	1	1	1	6	High
Kontochristopoulos et al., 2021 [48]	1	1	0	1	0	0	0	1	1	1	1	6	High
Kosova et al., 2022 [97]	1	1	0	1	0	0	0	1	1	1	1	6	High
Lee et al., 2020 [98]	1	1	0	1	0	0	0	1	1	1	1	6	High
Ojeda-Aravena, 2020 [9]	1	1	0	1	0	0	0	1	1	1	1	7	High
Redondo et al., 2014 [41]	1	1	0	1	0	0	0	1	1	1	1	6	High
Sannicandro et al., 2014 [99]	1	1	0	0	0	0	0	0	1	1	1	4	Moderate
Singh, 2012 [100]	1	1	0	1	0	0	0	1	1	0	1	5	Moderate

A detailed explanation for each PEDro scale item can be accessed at https://www.pedro.org.au/english/downloads/pedro-scale (accessed on 23 November 2022). ^a^ From a possible maximal score of 10.

**Table 4:** Participants characteristics and plyometric jump training programming variables.

	Sex	Age	BM	Height *	SPT	SpoP	Fit	Freq	Dur	Int	BH	NTJ	T	RBS	RBR	RBTS	PO	TP	Repl	Tap	Comb
Akın 2020	F-M	15–19	NR	NR	NR	TKD	Mod	3	6	NR	NR	NR	Mix	NR	NR	NR	NR	IS	No	No	No
al Syurgawi 2019	M	16.7	61.7	164	NR	Silat	Mod	2	6	NR	NR	NR	NR	NR	NR	≥48	Vo, T	NR	Yes	NR	No
Chaouachi 2014	M	11	40.1	150	No	WRT; judo	Mod	2	12	Max	NR	1080	V + B	3	NR	72	Vo	NR	Yes	Yes	No
Dallas 2020	F	13.9	48.9	160	NR	TKD	Mod	2	4	NR	NR	784	Mix	1.5	NR	≥48	Vo, T	NR	No	No	No
di Cagno 2020	M	17.6	64.2	173	Yes	Fen	High	2	6	NR	<50	60 min + 336	Mix	2	NR	72	Vo	IS	NA	No	No
Kontochristopoulos 2021	M	16	66.7	175	No	Fen	Mod-high	2	6	NR	NA	108	TJ	1–3	NR	72–96	No	PS	No	Yes	RT
Kosova 2022	NR	15.2	61.8–63.3	166–172	NR	Fen	Mod	3	8	NR	NA	2970	Mix	NR	NR	NR	Vo	NR	No	No	No
Lee 2020	M	22–24	69.6–66.6	172–173	NR	TKD	Mod	2	8	NR	NR	NR	Mix	0.5	NA	48	NR	NR	NR	No	No
Ojeda-Aravena 2020	M	15.6	63.3	167.3	NR	Karate	Mod	3	6	Max	NA	54–72 min	CMJ	2.5	NA	48	Vo	NR	Yes	No	No
Redondo 2014	M	24.8	70.4	173	Yes	Fen	High	2	6	Max	NA	432	V+ B + A	3	NR	≥48	No	IS	Yes	No	RT
Sannicandro 2014	F-M	13–14	NR	NR	NR	Fen	Mod	2	8	High	15–30	1600	Mix	1.5	45	NR	Vo, T	NR	Yes	No	LD
Singh 2012	M	14–15	NR	NR	NR	TKD	Mod	3	6	NR	NR	1350	Mix V	1	NR	NR	Vo	NR	NR	No	RT

A: acyclical; B: bilateral; BH: box or obstacle height (cm); BM: body mass (kg); Comb: exercises other than PJT were included (although PJT exercises represented ≥50% of the total training load); Dur: number of PJT weeks; Fen: fencing; Fit: fitness level (categorized as outlined previously [37]); Freq: number of PJT weekly sessions; Int: intensity of PJT; IS: in-season; LD: ladder drills (*quick feet* drills); Mod: moderate (includes competitive amateur athletes); NA: not applicable; Nor: normal; NR: not reported; NTJ: number of total jumps (e.g., repetitions); PO: progressive overload; Repl: athletes replaced part of their standard training with PJT; RBR: rest (s) between repetitions; RBS: rest (min) between sets; RBTS: rest (h) between sessions; RT: resistance training (*traditional* high-load slow-speed RT); SpoP: sport practiced; SPT: systematic PJT before the intervention; T: type of PJT drills; Tap: tapering, as a reduction in the number of jumps during the last week(s) of intervention; TJ: tuck jump; TKD: taekwondo; TP: training period of the season; V: vertical; Vo: volume (e.g., repetitions); WRT: wrestling; *: height in cm.

**Table 5:** Meta-analyses for CoSA physical fitness outcomes after PJT compared to control conditions.

Outcome	Studies *n*	E/C Groups *n*	Participant *n*	Effect Size	95% CI for Effect Size	*p* Value	I^2^
Maximal strength	4	4/4, 1 SA	117	0.77 (1.04) *	0.14 to 1.40 (0.46 to 1.62)	0.016 (<0.001)	60.0 (19.4)
Squat jump	3	3/3, 1 SA	72	0.06 (0.47)	−0.55 to 0.68 (−0.36 to 1.31)	0.837 (0.266)	29.8 (0.0)
Countermovement jump	7	7/7, 1 SA	167	0.69 (0.89)	0.01 to 1.36 (0.24 to 1.55)	0.045 (0.008)	76.8 (64.9)
CODS	3	3/3	46	0.72	0.16 to 1.28	0.012	0.0
Body mass	5	5/5, 1 SA	122	0.02 (0.02)	−0.33 to 0.36 (−0.43 to 0.47)	0.929 (0.932)	0.0 (0.0)
Fat mass	4	4/4	68	0.06	−0.39 to 0.51	0.801	0.0
Muscle mass	4	4/4, 1 SA	92	−0.01 (−0.06)	−0.40 to 0.38 (−0.64 to 0.53)	0.954 (0.851)	0.0 (0.0)
CoSA SP	3	3/3, 1 SA	86	0.35 (0.72)	−0.19 to 0.89 (0.04 to 1.40)	0.206 (0.038)	30.7 (0.0)

Abbreviations ordered alphabetically: CI: confidence interval; CODS: change of direction speed; CoSA: combat sport athlete; E/C: experimental/control; PJT: plyometric jump training; SA: specific–active, denoting a group of participants performing an alternative training-intervention (other than PJT); SP: specific performance (e.g., fencing movement velocity); *: all table values indicated in parenthesis denote those obtained after a sensibility analysis was performed, removing a study that incorporated a specific–active control group.

**Table 6:** Certainty of evidence for meta-analysis outcomes.

Outcome	N° Trials (n° Participants)	Comparisons	Certainty of Evidence
Maximal strength	4 (*n* = 117)	PJT versus specific-active (1 group) or active controls (3 groups)	Moderate ^b^
Squat jump	3 (*n* = 72)	PJT versus specific-active (1 group) or active controls (2 groups)	Moderate ^b^
Countermovement jump	7 (*n* = 167)	PJT versus specific-active (1 group) or active controls (6 groups)	Low ^a, b^
CODS	3 (*n* = 46)	PJT versus active controls (3 groups)	Moderate ^b^
Body mass	5 (*n* = 122)	PJT versus specific-active (1 group) or active controls (4 groups)	Moderate ^b^
Fat mass	4 (*n* = 68)	PJT versus active controls (4 groups)	Moderate ^b^
Muscle mass	4 (*n* = 92)	PJT versus specific-active (1 group) or active controls (3 groups)	Moderate ^b^
CoSA SP	3 (*n* = 86)	PJT versus specific-active (1 group) or active controls (2 groups)	Moderate ^b^

^a^—Downgraded by one level due to moderate impact of statistical heterogeneity (>25%). ^b^—Downgraded by one level due to <800 participants for the comparison or unclear direction of the plyometric jump training (PJT) effects. Evidence started at a high level of certainty (per outcome), but was downgraded based on the following criteria: (i) *Risk of bias in studies*: judgments were downgraded by one level if the PEDro scores for most of the studies were moderate (<6) or by two levels if they were poor (<4); (ii) *Indirectness*: low risk of indirectness was attributed by default due to the specificity of populations, interventions, comparators and outcomes being guaranteed by the eligibility criteria; (iii) *Risk of publication bias*: downgraded by one level if there was suspected publication bias; (iv) *Inconsistency*: judgments were downgraded by one and two levels when the impact of statistical heterogeneity (I^2^) was moderate (≥25%) or high (>75%), respectively; (v) *Imprecision*: one level of downgrading occurred whenever <800 participants were available for a comparison and/or if there was no clear direction of the effects. In case both were observed, certainty was downgraded by two levels. When the number of comparison trials was insufficient to perform meta-analysis, the evidence was automatically judged at very low certainty. Therefore, for the outcomes not included in the meta-analyses, the certainty of evidence should be considered very low.

## Data Availability

All data generated or analyzed during this study will be/are included in the published article as Table(s) and Figure(s). Any other data requirement can be directed to the corresponding author upon reasonable request.
